# Mapping the genetic landscape of hereditary diffuse-type gastric cancer progression

**DOI:** 10.1007/s10120-026-01730-1

**Published:** 2026-03-27

**Authors:** Lars J. S. Kemp, Remco van Cruchten, Robin Lomans, Natasja Rutgers, Lodewijk A. A. Brosens, Liudmila L. Kodach, Jolanda M. van Dieren, Tanya M. Bisseling, Richarda de Voer, Martijn Gloerich, Chella R. S. van der Post

**Affiliations:** 1 Center for Molecular Medicine, University Medical Center Utrecht, Utrecht, The Netherlands; 2 Department of Pathology, Radboud University Medical Center, Nijmegen, The Netherlands; 3 Department of Cancer Genomics, Radboud University Medical Center, Nijmegen, The Netherlands; 4 Department of Pathology, Netherlands Cancer Institute, Amsterdam, The Netherlands; 5 Department of Gastrointestinal Oncology, Netherlands Cancer Institute, Amsterdam, The Netherlands; 6 Department of Pathology, University Medical Center Utrecht, Utrecht, The Netherlands; 7 Department of Gastroenterology and Hepatology, Radboud University Medical Center, Nijmegen, The Netherlands

**Keywords:** Stomach neoplasms, Carcinoma, Signet ring cell, Cadherins, Exome sequencing

## Abstract

**Background:**

Hereditary diffuse gastric cancer (HDGC), caused by pathogenic variants (PVs) in *CDH1*, typically presents as early-stage mucosal lesions composed of non-proliferative signet ring cells (SRCs). While loss of E-cadherin initiates tumorigenesis, the somatic genetic alterations driving progression to advanced disease remain poorly understood.

**Objective:**

Our goal was to identify morphological and genetic changes associated with HDGC progression.

**Design:**

We performed whole exome sequencing on 38 gastric tumors from 26 HDGC patients, spanning early to advanced stages, and compared genetic alterations across tumor stages.

**Results:**

Early-stage HDGC lesions exhibited minimal somatic alterations, with low tumor mutational burden (TMB) and few cancer-related mutations. In contrast, advanced tumors showed a significant increase in TMB and frequent somatic mutations in *CDH1*, *TP53*, and genes involved in TGF-β signaling, cell adhesion, and actomyosin contractility (e.g., *SMAD4*, *FAT4*, *RHOA*). Copy number variations (CNVs) were also more prevalent in advanced tumors, including recurrent loss of 3p and 17p and amplification of oncogenes *MYC* and *MET*. CNV profiles varied between tumor regions, indicating intratumoral heterogeneity and potential clonal evolution.

**Conclusion:**

HDGC progression is marked by a stepwise accumulation of somatic mutations and chromosomal alterations. While early lesions remain genetically quiet, advanced tumors exhibit complex genetic landscapes, including *CDH1* inactivation, oncogenic mutations, and CNVs. These findings highlight key molecular events in HDGC progression and may inform future strategies for early detection and targeted intervention.

**Supplementary Information:**

The online version contains supplementary material available at 10.1007/s10120-026-01730-1.

## Introduction

Gastric cancer is the third leading cause of cancer-related deaths worldwide, with diffuse-type gastric cancer (DGC) having a worse prognosis compared to intestinal-type gastric cancer [1]. Prophylactically removed stomachs of hereditary DGC (HDGC) patients typically show multiple early-stage lesions, which are comprised mostly of non-proliferative, signet ring cells (SRCs). These differentiated SRCs display a characteristic morphology with their nucleus pushed to the side due to intracellular mucus accumulation. The presence of SRCs defines these lesions histopathologically as signet-ring cell carcinomas (SRCC), which are common in HDGC patients. Early-stage lesions are asymptomatic and restricted to the mucosal layer. Occasionally, mucosal lesions contain a higher proportion of poorly differentiated cells (PDCs) that are typically proliferative, and this likely represents the transitional stage (pT1a+) of DGC [2]. This is followed by a fraction of PDCs becoming invasive and infiltrating beyond the mucosa into the muscularis mucosa and deeper gastric layers [2]. The events driving this progressive development of advanced HDGC remain poorly understood.

The primary initiating event in both sporadic and hereditary DGC is the loss of functional E-cadherin protein. Germline pathogenic variants (PVs) in the encoding *CDH1* gene or related *CTNNA1* gene (encoding for αE-catenin) underlie genetic predisposition to HDGC [3, 4]. The majority of germline *CDH1* PVs in HDGC affect protein function due to truncations, large deletions or frameshifts [4]. In mouse models, formation of early-stage DGC depends on bi-allelic loss of *CDH1*, [5–8] which in HDGC patients is caused by somatic mutations in the second *CDH1* allele, loss of heterozygosity (LOH) or promotor methylation [9–12]. Homozygous loss of *CDH1* in the murine gastric epithelium results in formation of early-stage lesions, but this is insufficient for development of advanced DGC. Upon the presence of mutagenic agents, *TP53* loss, or infection, advanced DGC can develop, indicating that factors beyond *CDH1* may be necessary for progression into advanced DGC [8, 10, 13].

Spontaneously occurring somatic mutations in the gastric epithelium may represent additional oncogenic events driving advanced HDGC formation [14]. Currently, most of our knowledge on genetic drivers of advanced DGC depends on sequencing studies of sporadic DGC that revealed recurrent *TGFBR2*, *TP53* and *RHOA* mutations [15–18]. Whether these or other mutations occur during the development of advanced HDGC remains unknown. Moreover, the vast majority of sequenced sporadic DGC tumors have been of advanced stage, and thus, genetic differences between early, transition and advanced stages of DGC remain uncharacterized. As a result, the genetic alterations contributing to the stepwise progression from early-stage disease to advanced HDGC in the presence of germline PVs in *CDH1* remain unexplored.

The presence of early-stage lesions in prophylactically resected stomachs from HDGC patients provides the opportunity to explore the genetic changes that contribute to the initiation and progression towards advanced HDGC. In this study, we explore the genetic landscape of the different stages of HDGC to uncover the somatic events that contribute to disease progression.

## Materials and methods

### Human tissue samples

Formalin-fixed paraffin-embedded (FFPE) tissue samples were collected from 38 DGC lesions, coming from 26 patients harboring a confirmed germline *CDH1* PV, from the pathology archives of Radboud university medical center (Radboudumc), Antoni van Leeuwenhoek Hospital, and UMC Utrecht (Table 1). The study received approval from all three institutional review boards, with Radboudumc serving as the lead center (approval number 2020-6617). A waiver of consent was granted for this study, ensuring that data from patients who opted out of research use were excluded.

**Table 1:** Clinical and Sample Characteristics of HDGC Cases Selected for Whole Exome Sequencing

Patient	Germline pathogenic variant in *CDH1*	Sex (M/F)	Resection type: curative gastrectomy (CG), prophylactic gastrectomy (PTG), biopsy (Bx), metastasis (M)	Age group (years)	Sample_id: normal (N), tumor (T)	pTN-stage	Tissue selection of tumor: Superficial layer (SL), deeper layers (DL), metastasis (MET)
1	c.1135_1137 + 5delinsTTAGA	M	CG	51–55	N1	pT3N3a	Normal
					T1-A	pT3N3a	SL
					T1-B	pT3N3a	DL
					T1-C	pT3N3a	DL
2	c.1135_1137 + 5delinsTTAGA	F	CG	31–35	N2	pT3N0	Normal
					T2	pT3N0	DL
3	c.1135_1137 + 5delinsTTAGA	F	CG	26–30	N3	pT4bN1	Normal
					T3-A	pT4bN1	SL
					T3-B	pT4bN1	DL
					T3-C	pT4bN1	DL
					T3-D	pT4bN1	MET
4	c.2195G > A; p.(Arg732Gln)	F	Bx	36–40	N4	pTxNx	Normal
					T4	> pT3Nx	SL
5	c.1476_1477del; p.(Arg492fs)	M	CG	41–45	N5	pT4aN1	Normal
					T5-A	pT4aN1	SL
					T5-B	pT4aN1	DL
					T5-C	pT4aN1	DL
6	c.1476_1477del; p.(Arg492fs)	F	M	26–30	N6	pTxNxM1	Normal
					T6	pTxNxM1	MET
7	c.2064_2065del; p.(Cys688*)	M	CG	46–50	N7	pT3N2M1	Normal
					T7-A	pT3N2M1	SL
					T7-B	pT3N2M1	SL
					T7-C	pT3N2M1	DL
					T7-D	pT3N2M1	DL
					T7-E	pT3N2M1	MET
8	c.377del; p.(Pro126fs)	M	CG	36–40	N8	pT3N0	Normal
					T8-A	pT3N0	SL
					T8-B	pT3N0	DL
					T8-C	pT3N0	DL
					T9	pT1aN0	SL
9	c.1565 + 2dup	F	CG	36–40	N9	pT4aN2	Normal
					T10-A	pT4aN2	SL
					T10-B	pT4aN2	DL
					T10-C	pT4aN2	DL
10	c.811_812delinsTTAAGGGATATA; p.(Val271fs)	F	CG	36–40	N10	pT4aN0	Normal
					T11	pT4aN0	DL
11	CDH1 c.163 + 2 T > A p.? (p.?)	F	CG	46–50	N11	ypT1aN0	Normal
					T12	ypT1aN0	SL
12	CDH1 c.163 + 2 T > A (p.?)	M	CG	51–55	N12	pT3N0	Normal
					T-13	pT3N0	DL
13	CDH1 c.971del (p.(Gly324fs))	F	PTG	21–25	N13	pT1aN0	Normal
					T14	pT1aN0	SL
					T15	pT1aN0	SL
14	c.2195 G > A (p.(Arg732Gln))	M	PTG	21–25	N14	pT1aN0	Normal
					T16	pT1aN0	SL
					T17	pT1aN0	SL
					T18	pT1aN0	SL
15	c.1565 + 2dup	F	CG	46–50	N15	pT4aN2M1	Normal
					T19-A	pT4aN2M1	SL
					T19-B	pT4aN2M1	DL
					T19-C	pT4aN2M1	DL
16	c.2195G > A (p.(Arg732Gln)	M	PTG	31–35	N16	pT1aN0	Normal
					T20	pT1aN0	SL
					T21	pT1aN0	SL
17	c.(?_-1)_(1320 + 1_1321-1)del (deletion exon 1 t/m 9)	F	PTG	36–40	N17	pT1aN0	Normal
					T22	pT1aN0	SL
					T23	pT1aN0	SL
18	c.(?_-1)_(1320 + 1_1321-1)del (deletion exon 1–9)	F	PTG	36–40	N18	pT1aN0	Normal
					T24-A	pT1aN0	SL
					T24-B	pT1aN0	SL
19	c.1792C > T p.(Arg598*)	F	PTG	51–55	N19	pT1aN0	Normal
					T25	pT1aN0	SL
					T26	pT1aN0	SL
					T27	pT1aN0	SL
					T28	pT1aN0	SL
					T29	pT1aN0	SL
20	c.187C > T (p.(Arg63*))	M	PTG	21–25	N20	pT1aN0	Normal
					T30	pT1aN0	SL
21	(c.l565-t-lG > A)	M	PTG	46–50	N21	pT1aN0	Normal
					T31	pT1aN0	SL
22	c.1135_1137 + 5delinsTTAGA	M	PTG	21–25	N22	pT1aN0	Normal
					T32	pT1aN0	SL
					T33	pT1aN0	SL
					T34	pT1aN0	SL
23	c.163G > A p.(Val55*)	F	PTG	56–60	N23	pT1aN0	Normal
					T35	pT1aN0	SL
24	c.(832 + 1_833-1)_(1320 + 1_1321-1)dup (duplication exon 7–9)	F	PTG	21–25	N24	pT1aN0	Normal
					T36	pT1aN0	SL
25	c.1135_1137 + 5delinsTTAGA	F	PTG	16–20	N25	pT1aN0	Normal
					T37	pT1aN0	SL
26	c.(832 + 1_833-1)_(1320 + 1_1321-1)dup (duplication exon 7–9)	M	PTG	46–50	N26	pT1aN0	Normal
					T38	pT1aN0	SL

M, male; F, female; CG, curative gastrectomy; PTG, prophylactic gastrectomy; Bx, biopsy; M, metastasis; N, normal; T, tumor; pTN-stage, pathological tumor-node stage; SL, superficial layer; DL, deeper layers; MET, metastasis; c., coding DNA; p., protein; del, deletion; dup, duplication; delins, deletion–insertion; fs, frameshift; *, stop codon

### Histology and immunohistochemistry on patient tissue sections

Immunohistochemistry was performed using the Dako Omnis platform (Agilent Technologies) using an automated staining protocol. Slides were stained for E-cadherin with mouse anti-E-cadherin (NCH-38, Dako Omnis, GA059; FLEX Ready-to-use IHC) and classified as loss of stain, weak to moderate (irregular) membranous staining or strong (irregular) membranous staining. Haematoxylin and eosin (H&E) and E-cadherin stained tissue sections were scanned using a Pannoramic 1000 DX slide scanner (3DHistech) using a 20 × objective or 40 × optical equivalent magnification.

### Detection of cell-types

A deep learning model was used to detect and count the number of typical signet ring cells (SRCs), smaller and/or irregular SRC cells and poorly differentiated cells (PDCs) in H&E-stained HDGC lesions. This model was previously trained and validated on an annotated multi-centre dataset of digitized histopathology images from four European medical institutions [19].

### Whole exome sequencing

1–10 sections of 10 µm (depending on size of the tumor area) were cut and macro-dissected to isolate tumor regions pre-selected by a pathologist. DNA was isolated via the Chelex-100 procedure, after which DNA was mechanically sheared on a Covaris R230 ultrasonicator and library preparation was performed using Twist Library Preparation Kit with Amp Mix for Mechanical Fragmentation (Twist Bioscience) reagents. Whole exome sequencing (WES) was performed via enrichment with Twist Comprehensive Exome 2.0 probes and sequencing on an Illumina NovaSeq6000, aiming at 400 × coverage per sample. Data-analysis was performed with an inhouse bio-informatic pipeline relying on bwa-mem2 for alignment, Mutect2 for variant calling and PureCN for copy-number calling [20–22]. See Supplementary Methods for details on Somatic variant calling and Mutational signature analysis. Whole exome sequencing data have been deposited in the EGA and are available under the Data Access Committee: Radboudumc Cancer Genomics DAC (EGAC00001001102).

## Results

### Sequencing of HDGC lesions that include different cancer cell type populations

**Fig. 1:**
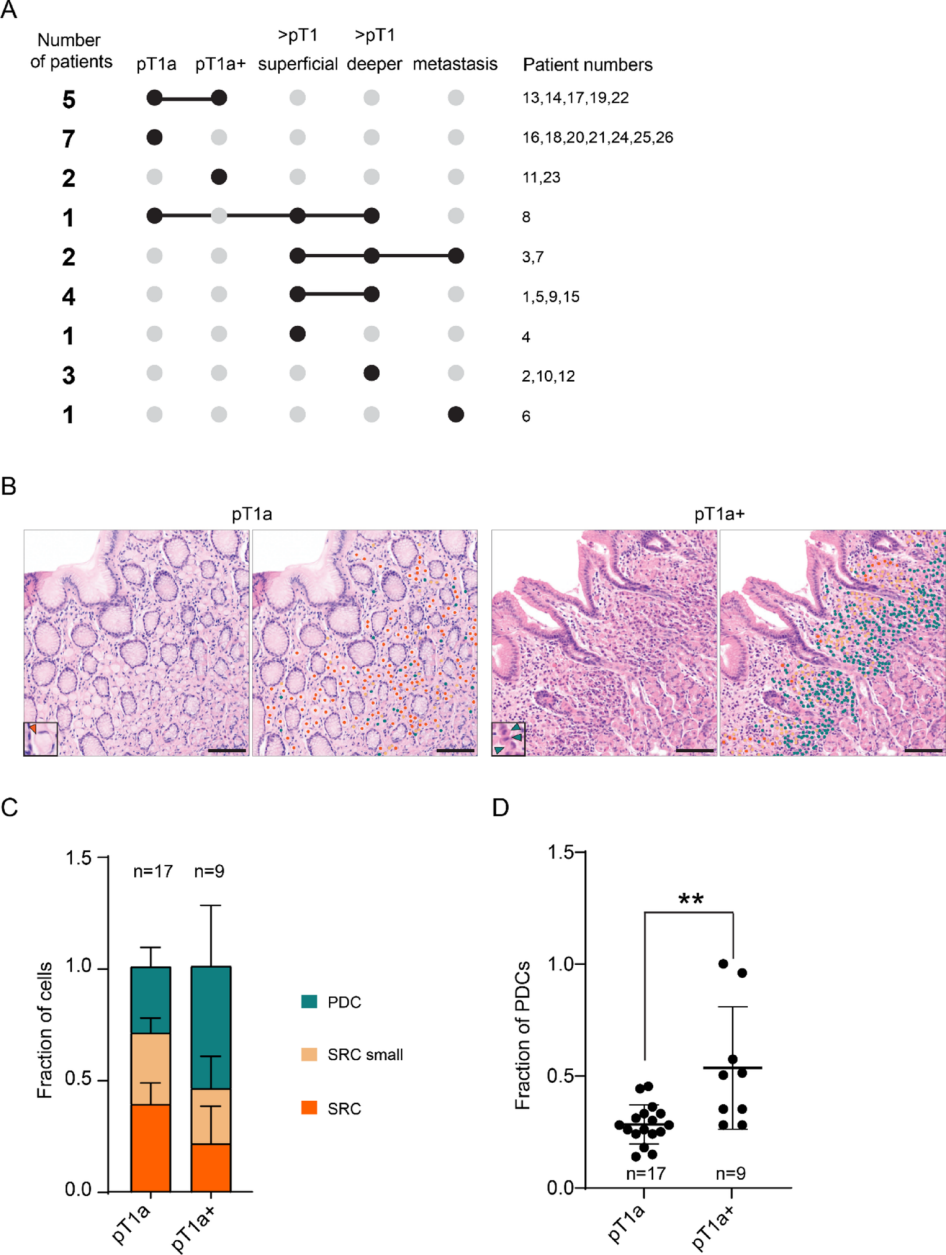
Overview of HDGC samples and histopathological analysis of early-stage lesions. **A** Overview of hereditary diffuse gastric cancer (HDGC) samples included in this study. Connected lines indicate samples of different HDGC stages from the same patient. **B** Representative Hematoxylin and eosin (H&E) staining of pT1a and pT1a+ HDGC lesion with color-marked cell populations; signet ring cells (SRC, orange), small SRCs (light-orange) and poorly differentiated cells (PDCs, blue). Scalebar represents 100 µm. **C** Quantification of the relative proportions of SRCs, small SRCs, and PDCs in pT1a versus pT1a+ lesions. **D** Quantification of the relative proportion of PDCs in pT1a versus pT1a+ lesions. Mann–Whitney test, **p < 0.005

To uncover whether genetic changes occur during progression towards advanced malignant HDGC, we collected 38 HDGC tumors of different stages (Fig. 1A) from 26 patients, as well as tissue from surrounding intact glands. We included 12 advanced HDGC tumors that infiltrated beyond the muscularis mucosa and 3 metastases (2 lymph node, 1 ovarian). For advanced tumors, we analyzed separate regions, including 9 samples from the mucosal side (superficial layer) and 17 from regions that infiltrated beyond the submucosa of the gastric wall (deeper layer) (Table 1). Additionally, we included 26 early-stage lesions confined to the mucosa.

Early-stage HDGC lesions were morphologically heterogeneous. Many pT1a lesions consisted predominantly of classic, well-differentiated SRCs but occasionally showed increased proportions of atypical SRCs and PDCs, loss of spatial separation of SRC subtypes, or stromal reaction. Based on these features, such lesions were classified as atypical mucosal lesions (pT1a+), representing a proposed transition stage towards advanced HDGC [23]. Quantification confirmed a significant increase in PDCs in pT1a+ lesions compared to pT1a lesions (Fig. 1B–D). Altogether, this sampling strategy enabled comparative analysis of genetic changes associated with HDGC progression.

### Somatic mutations in *CDH1* across HDGC stages

**Fig. 2:**
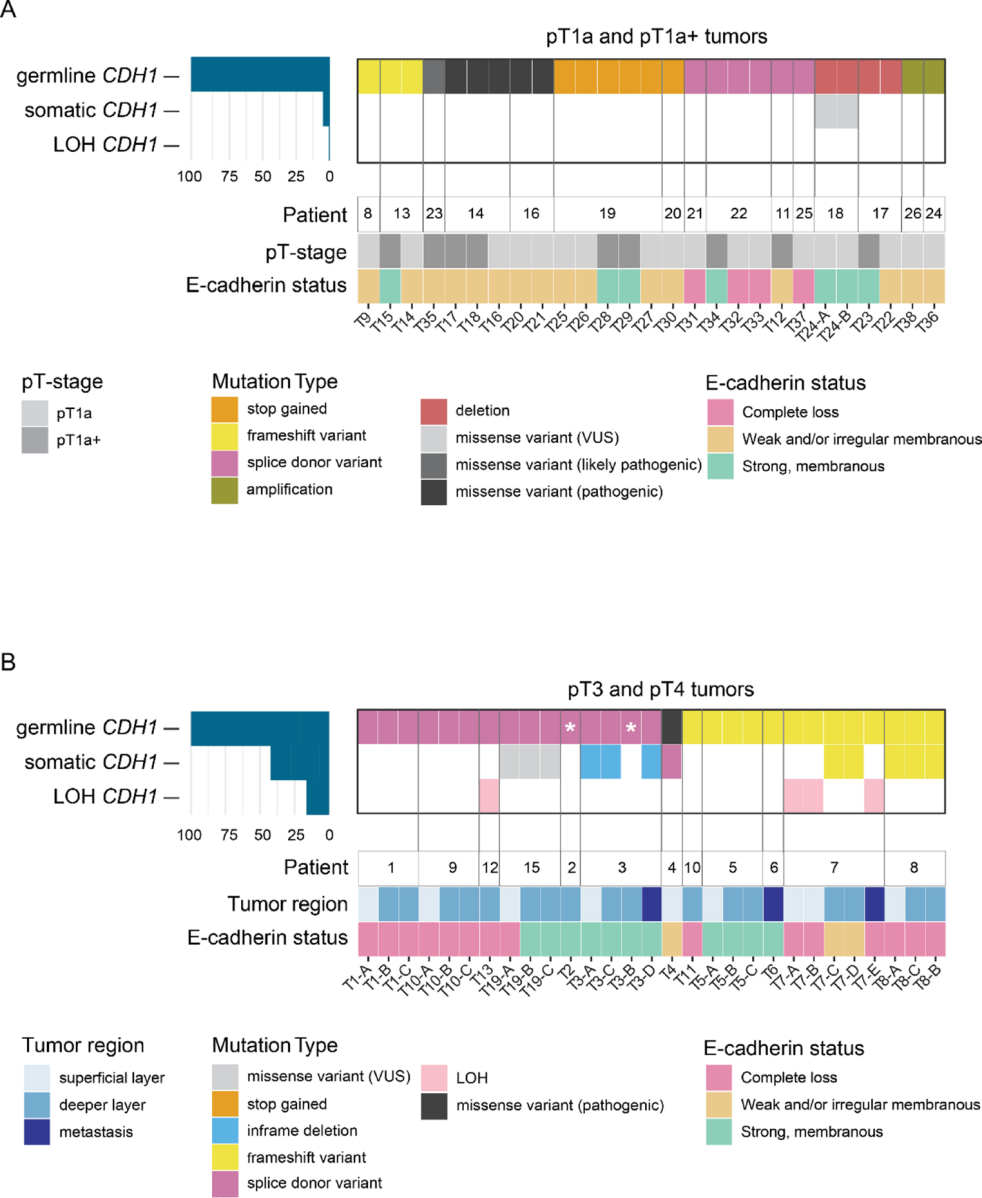
Mutational landscape of *CDH1* in early and advanced HDGC lesions. Oncoplot illustrating germline and somatic *CDH1* mutations, as well as loss of heterozygosity (LOH), in early-stage HDGC samples (**A**) and advanced HDGC (**B**). Mutational annotations within the oncoplot include color-coded variant type as defined in the figure legend. For both plots, accompanying bars underneath indicate the tissue origin, stage and lesion depth for each sample, with a corresponding legend provided. Furthermore, the status of E-cadherin distribution determined by immunohistochemistry is indicated (completely absent; weak and/or irregular membranous; strong regular staining). Grey vertical lines demark samples derived from the same patient. White asterisks indicate samples of which the germline PV was detected in normal gastric glands, but could not be identified in the corresponding tumor sample

We performed whole exome sequencing on the 56 samples derived from 38 HDGC tumors. In tissue from every patient, we could identify the germline PV in *CDH1* (Table 1). To test whether genetic changes underly loss of the second allele of *CDH1* in our tumor set, we analyzed the occurrence of loss of heterozygosity (LOH) and somatic mutations. In early (pT1a and pT1a+) lesions, we could only confirm a second hit in one tumor (somatic mutation 1/26, 3.8%) (Fig. 2A, supplementary Tables 1, 2). In contrast to early-stage lesions, advanced HDGC more frequently harbored somatic *CDH1* mutations (5/12, 41.7%) and LOH (2/12; 16.7%) (Fig. 2B, supplementary Tables 1, 2). While the majority of advanced HDGC cases lacked a wild-type *CDH1* allele due to somatic alterations (6/12; 50%), in most early-stage HDGC tumors we did not detect somatic events affecting *CDH1* (1/26; 3.8%) (Fisher’s exact test, P = 0.002). These findings suggest stage-specific differences in second-allele alterations of *CDH1*, although low tumor percentage may have limited our ability to detect such events in early lesions.

We further performed immunohistochemistry to assess the expression pattern of E-cadherin across lesions, identifying three main staining patterns: complete loss (*n* = 18, 32%), weak to moderate membranous staining (*n* = 19, 34%), or strong membranous staining (*n* = 19, 34%) (Figs. 2A, B and Supplementary Fig. 1A). The latter pattern of strong membranous staining, with minor irregularities, was indistinguishable from the normal membranous staining observed in non-neoplastic tissue. Most typical pT1a lesions (9/12, 75%) exhibited weak to moderate membranous staining, with only 1 out of 12 (8,3%) showing retained strong membranous expression (Fig. 2A and Supplementary Fig. 1B, C). Atypical pT1a+ lesions (4/7, 57%) and tumor regions in advanced lesions (7/17, 41%) more frequently retained strong membranous E-cadherin expression at the plasma membrane (Figs. 2A, B and Supplementary Fig. 1B, C). Nearly all advanced lesions displayed either strong membranous staining or complete loss of E-cadherin, with the latter correlating with the presence of frameshift mutations or LOH of *CDH1* (Fig. 2B and Supplementary Fig. 1B). Altogether, these findings delineate alterations of the second *CDH1* allele at different HDGC stages, which may underlie differences in E-cadherin expression status.

### Mutational load increases during HDGC progression

**Fig. 3:**
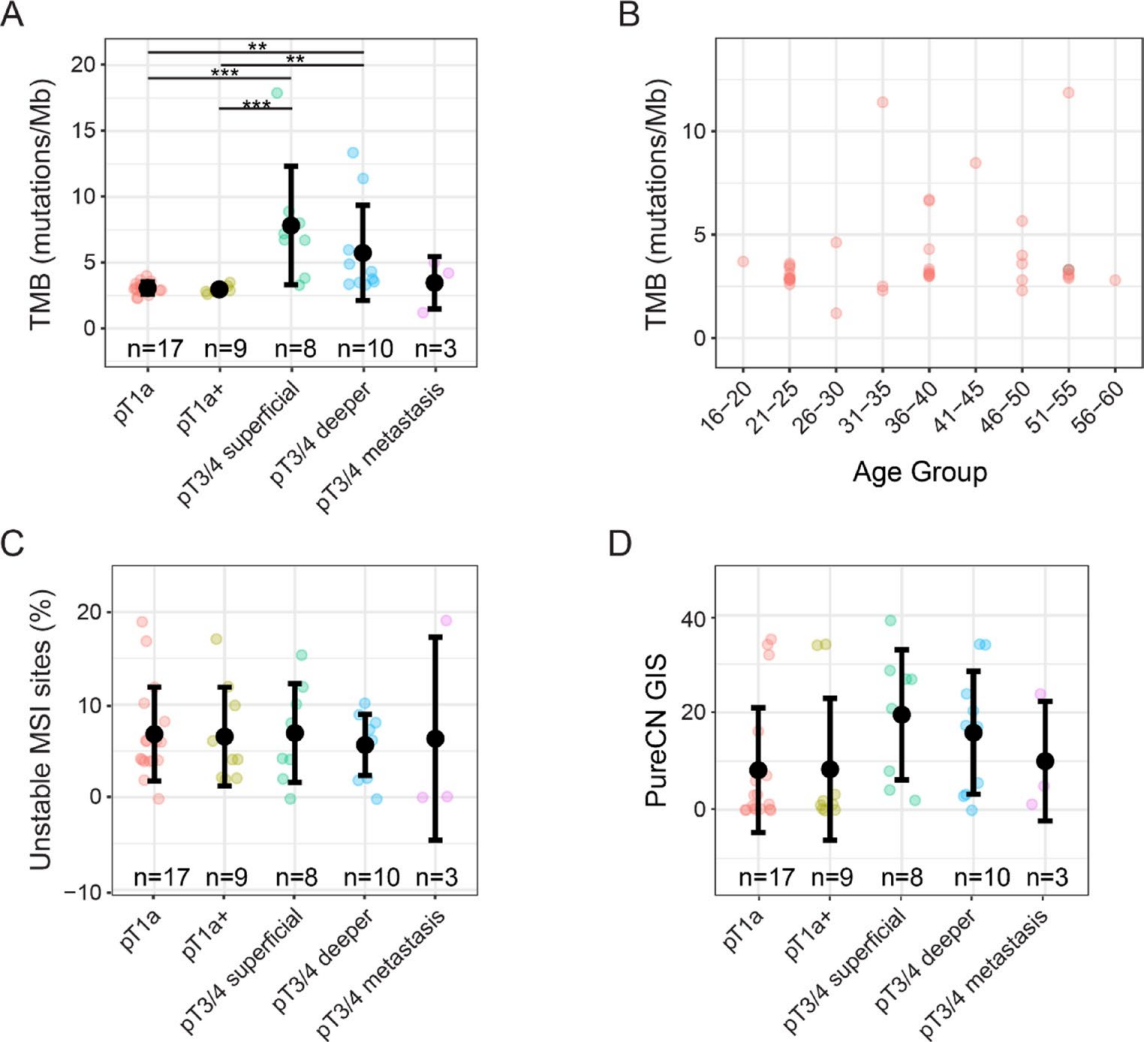
Somatic mutational burden across HDGC stages and age groups. **A** Dot plot showing the distribution of somatic mutational burden across different pathological stages of HDGC. Each dot represents the average score of a tumor region of an individual tumor. Dunn’s test, **p < 0.01, ***p < 0.005. **B** Scatter plot of binned age at diagnosis (grouped in 5-year intervals) versus somatic mutational burden. No correlation was observed between age and mutation count, suggesting age is not a primary driver of mutational load. **C** Dot plot showing the distribution of the percentage of unstable MSI sites as determined by PureCN across different pathological stages of HDGC. Each dot represents the average score of a tumor region of an individual tumor. Dunn’s test, all not significant. **D** Dot plot showing the distribution of the GIS-score as determined by PureCN across different pathological stages of HDGC. Each dot represents the average score of a tumor region of an individual tumor. Dunn’s test, all not significant

Next, we aimed to uncover somatic genetic alterations, beyond *CDH1* mutations, that occur in different progressive stages of HDGC. First, we determined the tumor mutational burden (TMB) across all stages. Early-stage tumors showed a relatively low TMB with minimal differences between pT1a and pT1a+ tumors, which on average had a TMB of 3.1 and 3.0 mutations/Mb, respectively (Fig. 3A). In advanced tumors (pT3/4) the mutational burden doubled to an average TMB of 7.8 mutations/Mb in the superficial layer and 5.7 in the deeper layer (Fig. 3A). Interestingly, the observed TMB of metastases was comparable to early-stage tumors (Fig. 3A). The increase of mutations in advanced tumors did not correlate with age (Fig. 3B). Additionally, while advanced HDGC exhibited a higher TMB, this increase was not associated with microsatellite instability (MSI), as no cases demonstrated MSI-high status (Fig. 3C). Furthermore, in accordance with previous published data, DGC lesions across all stages did not show clear evidence of genomic instability, as indicated by a Genomic Instability Score (GIS) below 50 and low frequencies of NTAI (Number of Telomeric Allelic Imbalances), LOH (Loss of Heterozygosity), and LST (Large-Scale Transitions) (Fig. 3D, Supplementary Fig. 2A) [24].

To explore whether specific mutagenic processes could potentially underlie the accumulation of somatic mutations, we investigated the presence of mutational signatures. This analysis may enable the discovery of the origin of somatic mutations based on the mutational profile. In total, 10 of our samples met our predetermined criteria for mutational signature analysis (see *Methods*). The mutational pattern correlated with signatures associated with age-related acquisition of mutations (SBS1) (Supplementary Fig. 2B). Furthermore, we observed a correlation with alkylating agents (SBS11) and immunosuppressive agents (SBS32) (Supplementary Fig. 2B). Included patients were not exposed to treatment regimens typically associated with these signatures, suggesting that these signatures may be caused by unknown endogenous processes that mimic their mutational footprint or represent a technical artifact.

### Mutations associated with HDGC progression

**Fig. 4:**
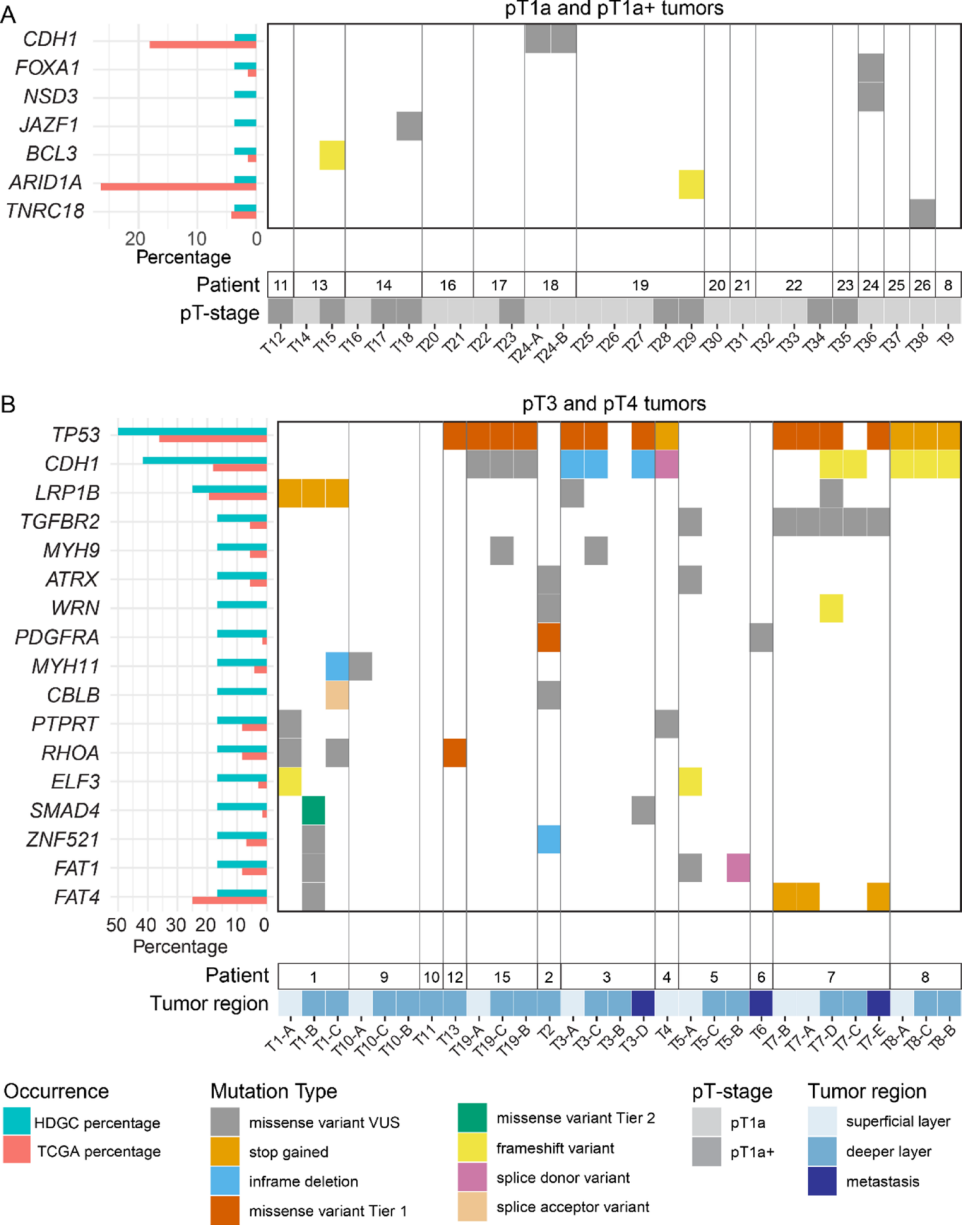
Mutational landscape of somatic mutations in early and advanced HDGC. Oncoplot illustrating the somatic mutations in early-stage HDGC samples (**A**) and advanced HDGC (**B**) ranked by the rate of occurrence in tumors. Mutational annotations within the oncoplot include color-coded variant type as defined in the figure legend. For both plots, accompanying bars underneath indicate the tissue origin, stage and lesion depth for each sample, with a corresponding legend provided. Grey vertical lines demark samples derived from the same patient

Next, we sought to identify and compare the somatic gene mutations in early-stage HDGC and advanced HDGC to identify genetic alterations that potentially underlie HDGC progression. In a subset of early lesions, we found somatic mutations in genes related to cancer development (Fig. 4A, supplementary Tables 3, 4), with no significant difference in the number of cancer-related somatic mutations between pT1a (1/17, 5.9%) and pT1a+ (3/9, 33.3%) tumors (Fisher’s exact test, *P* = 0.0933). Most somatic mutations were missense variants of uncertain significance, affecting *FOXA1*, *NSD3*, *JAZF1* and *TNRC18.* In two pT1a+ tumors, we identified frameshift mutations in the *BCL3* and *ARID1A* gene. Thus, while the majority of early lesions did not harbor any cancer-related somatic mutations, we identified two frameshift mutations in pT1a+ lesions that could potentially contribute to early tumor progression.

In advanced HDGC, *TP53* was the most frequent somatically mutated gene (6/12, 50%), more frequent than somatic mutations of *CDH1* (5/12, 41.7%) (Fig. 4B, supplementary Tables 3, 5). Tumor samples carrying *TP53* mutations showed a significantly higher percentage of unstable MSI sites, although the MSI status (median 11 [range 0–19] compared to median 1 [range 0–4] in *TP53*-wildtype) and GIS-score (median 26 [range 5–39] compared to median 3 [range 1–9] in *TP53*-wildtype) remained low, and TMB was comparable to advanced HDGC samples with wildtype *TP53* (Supplementary Fig. 3A–F). In addition to *TP53*, mutations occurred in genes encoding for components of the TGF-β signaling pathway (*SMAD4* and *TGFBR2*), adhesion proteins (*FAT1* and *FAT4*) and regulators of actomyosin contractility (*RHOA*, *MYH9* and *MYH11*). Identified mutations were generally present in both the superficial layer and the deeper invasive part of advanced HDGC. Similarly, the genetic pattern of metastases was comparable to their matched primary tumor samples. Thus, advanced HDGC harbors an increased number of cancer-associated somatic mutations compared to early lesions, which may influence signaling pathway activity and cellular function.

To assess whether similar genetic alterations occur in HDGC as in sporadic DGC, we compared the frequency of somatic mutations in our panel to that of sporadic tumors in TCGA (72 samples) (Fig. 4B, supplementary Table 6) [18]. For most genetic mutations in HDGC, the frequency was within a similar range as sporadic DGC, including mutations in *LRP1B*, *TP53*, *RHOA* and *FAT4*. However, a subset of genes was solely mutated in HDGC (21/98), including *WRN* and *CBLB,* and we observed an increase (albeit non-significant) in the number of somatic *CDH1* mutations in advanced HDGC (5/12, 41.2%) compared to sporadic DGC (13/72, 18.1%). Conversely, several genes that are frequently mutated in sporadic diffuse gastric cancer (DGC), such as *PIK3CA* (19.4%, 14/72) and *KMT2D* (16.7%, 12/72), remained unaltered in our cohort of HDGC tumors. Thus, although sporadic DGC and HDGC display similarities in their mutational pattern, the presence of germline PVs in *CDH1* may influence the genetic landscape, rendering it distinct from that of sporadic DGC.

### Recurrent chromosomal aberrations correlating with HDGC progression

**Fig. 5:**
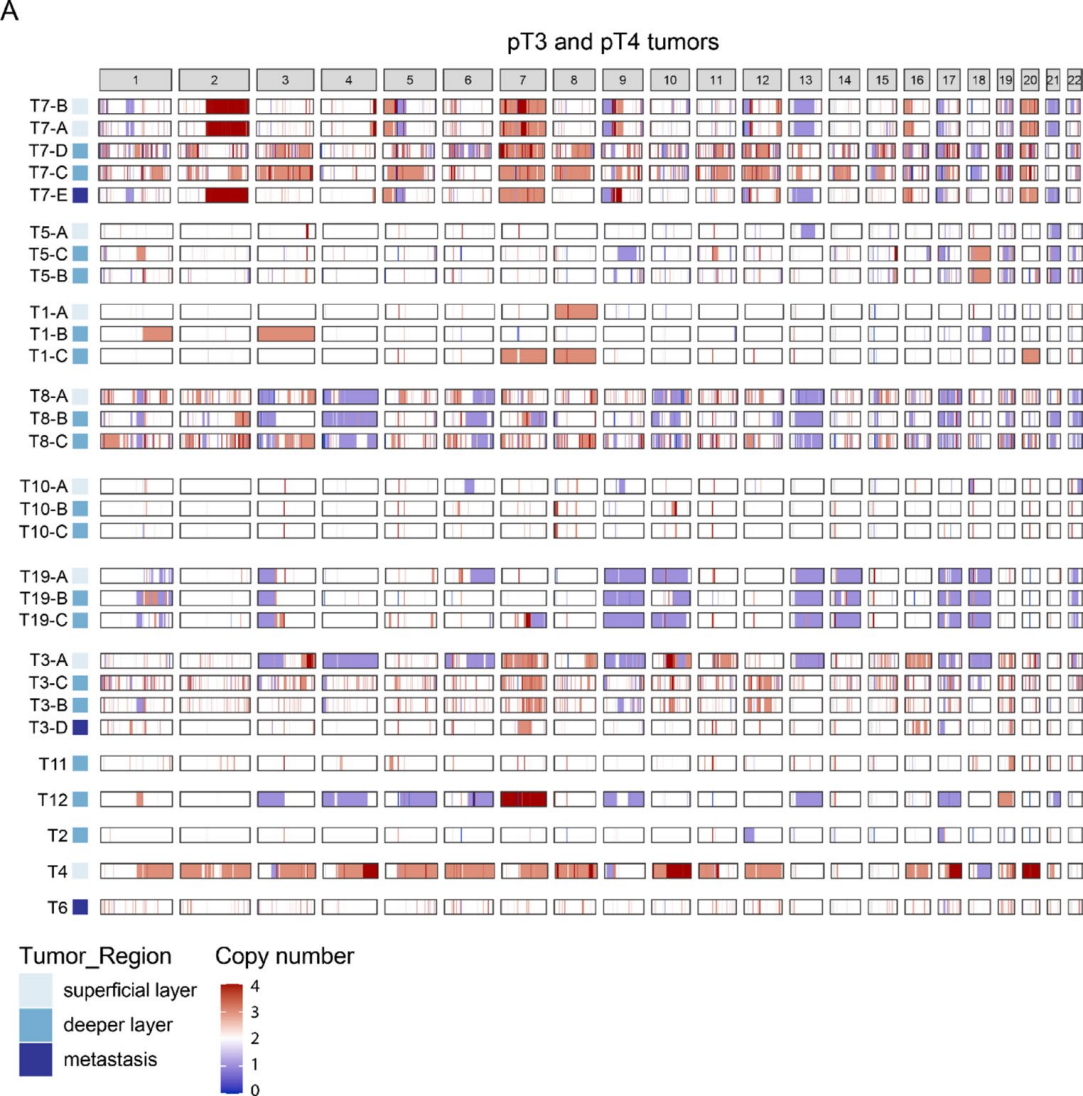
Recurrent genomic aberrations in advanced HDGC. Visualization of the absolute total copy number values provided by PureCN of pT3/4 tumors

Large-scale chromosomal rearrangements are prevalent in many cancers and have been consistently associated with poor prognosis [25]. These large chromosomal amplifications and deletions (aneuploidies) can display recurrent patterns within cancer subtypes. The Copy Number Variation (CNV)-pattern of early-stage HDGC tumors showed a low number of chromosomal amplification or deletions (Supplementary Fig. 4). In contrast, advanced HDGC tumors showed several recurrent chromosomal amplifications and deletions (Fig. 5). The CNV profiles exhibited heterogeneity across tumors, with some tumors (e.g., tumors 5 and 10) displaying limited number of aneuploidies, whereas others (e.g., tumors 7 and 8) showed multiple chromosomal copy number alterations. Furthermore, chromosomal copy numbers varied between samples obtained from distinct regions within the same tumor (e.g., chromosome 13 in tumors 3 and 7). Several chromosomal amplifications and deletions occurred in multiple tumors, including (i) loss of chromosome arm 3p (5/12 tumors) previously linked to reduced immune-response and increased epithelial-to-mesenchymal transition (EMT) gene expression [2, 25, 26]; (ii) loss of Chromosome 17 harboring tumor suppressor gene *TP53* (5/12 tumors); (iii) additional copies of chromosome 7 (5/12 tumors) and 8 (3/12 tumors), both chromosomes previously reported to be (partially) amplified in sporadic DGC [27]. In line with the CNV-changes (Fig. 5), amplifications can be observed of genes on chromosome 7 and 8, including well-known oncogenes *MYC* (7q31.2) and *MET* (8q24.13) respectively (supplementary Table 7). Thus, advanced HDGC tumors show distinct CNV-patterns, which further varies between different regions of individual tumors.

## Discussion

By sequencing HDGC tumors with a gPV in *CDH1*, we mapped genetic events from indolent pT1a to atypical pT1a+ to advanced DGC infiltrating the underlying deeper gastric layers and forming metastatic disease. Our analysis revealed minimal genetic changes in early-stage pT1a and pT1a+ lesions DGC, with an accumulation of mutational burden in advanced HDGC. Genetic alterations in advanced DGC include numerous non-synonymous mutations in genes that may affect a range of cellular processes, including actomyosin contractility and TGF-β signaling. Additionally, we show that advanced HDGC tumors show bias for acquiring amplifications of chromosomes harboring known oncogenes.

Previous reports identified hypermethylation, LOH, and somatic mutations as mechanisms underlying the inactivation of the functional wildtype *CDH1* allele [10–12]. We observed relatively few early-stage lesions with LOH or somatic *CDH1* mutations, whereas these alterations were significantly more frequent in advanced HDGC (Fig. 2). While promotor hypermethylation may initially silence *CDH1*, epigenetic reversibility could permit re-expression during progression, whereas LOH and somatic mutations lead to permanent loss of E-cadherin function. The enrichment of irreversible *CDH1* alterations in advanced tumors may reflect a selective advantage of permanent E-cadherin loss during DGC progression. This is supported by mouse models showing that retention of a functional *CDH1* allele halters advanced DGC formation [8, 29]. However, we cannot exclude that lower tumor cell percentages of early stage lesions hampered detection of somatic *CDH1* mutations. Furthermore, recent histochemical analyses suggest that E-cadherin is re-expressed in advanced DGC [30], and we similarly observed retained E-cadherin expression within the subset of advanced lesions lack somatic frameshift mutations or LOH of *CDH1* (Fig. 2B). These findings warrant integrated analysis of the epigenetic and genetic status of the *CDH1*-allele across tumor stages. Such studies may help to elucidate potential re-expression of E-cadherin during tumor evolution, which may be driven by changes in the local tumor microenvironment, and its potential association with metastasis.

Early-stage tumors (pT1a and pT1a+) displayed low frequency of somatic mutations, including 4 missense mutations of unknown significance. Notably, two pT1a+ lesions harbored frameshift mutations in cancer-related genes, potentially contributing to their more advanced morphology (Fig. 4). This includes a mutation in *ARID1A,* a component of SWI/SNF complexes that control chromatin remodeling, which has previously been identified in a substantial number of advanced DGCs [31–33]. ARID1A loss is thought to promote epigenetic dysregulation favorable to tumor progression [33, 34]. Together with mutations identified in other epigenetic regulators (*NSD2* in pT1a, *KAT6A* and *EP300* in advanced HDGC), this suggests possible contributions of epigenetic alterations during DGC development. The absence of somatic oncogenic mutations in most pT1a+ lesions implies that non-genetic mechanisms may drive the accumulation of PDCs at this stage. In line with this, previous work demonstrate that stromal extracellular matrix remodeling influences the differentiation status of DGC cells [35]. Nonetheless, insufficient sequencing depth or low mutant allele fractions in early lesions may have limited detection of driver events, which could be resolved by spatial genomic approaches [36, 37].

Advanced HDGC displayed substantially increased genetic alterations compared to early-stage lesions, with *CDH1* and *TP53* being the the most frequently somatically mutated genes (Fig. 4B). *TP53* mutations are associated with genomic instability, consistent with higher genomic instability scores in *TP53*-mutant advanced tumors. We further identified mutations that may affect the activity of signaling pathways implicated in tumor progression, including mutations in *SMAD4* and *TGFBR2* that may disrupt TGF-β signaling. In murine models harboring *CDH1* and *TP53* mutations, loss of *SMAD4* or *TGFBR2* mutations promoted tumor growth and metastasis [15, 38]. Mutations in *RHOA* and other actomyosin-related genes were also common. Attenuation of actomyosin contractility in advanced HDGC may enable resistance to apoptosis that could otherwise be induced by loss of adhesion caused by *CDH1* mutations [39, 40]. Although mutations in Wnt pathway components are uncommon in DGC, we identified recurrent mutations in *LRP1B* that is implicated in Wnt pathway regulation in gastric cancer progression [35, 41, 42]. While sporadic and hereditary DGC share mutational similarities, certain genes were uniquely affected in each (Fig. 4). This suggests that *CDH1* germline variants may shape the mutational trajectory of HDGC, and that the sporadic and hereditary form may depend on separate molecular mechanisms of progression.

In advanced HDGC, we observed genetic mutations to be similarly present in superficial and deeper layers of the tissue, suggesting no additional alterations are acquired during tumor invasion. This implies that prognostically or diagnostically relevant genetic alterations are distributed throughout the tumor, making them reliably detectable in superficial biopsies. Likewise, our analysis did not identify genetic selection during metastasis formation, as has previously been shown for *TGFBR2* mutations [15]. Although we did not detect intratumoral enrichment of specific genetic alterations, the OpCNV profiles (Fig. 5) imply that superficial layers are genetically more diverse than their infiltrative counterparts. This raises the possibility that multiple genetically distinct clones exist in the tumor with only select clones gaining invasive capacity. However, we cannot exclude that confounding factors, such as the tumor cell purity, explains observed differences in mutational burden.

Chromosomal copy number remains largely stable in early-stage HDGC, but recurrent amplifications and deletions emerged in advanced stages. While somatic mutations show limited intratumoral heterogeneity, CNV patterns vary across tumor regions, suggestive of selective pressures in specific microenvironments. Some of these alterations are linked to loss of tumor suppressors, EMT initiation, and enhanced growth signaling [26]. In line with this, we observed amplification of key oncogenes such as *MYC* and *MET* in advanced HDGC, both associated with poor prognosis and with potential for therapeutic targeting [28, 43–47]. Although GISTIC is used to assess recurrent CNVs, our sample size did not meet its requirements [48].

Here, we identified genetic alterations across progressive DGC stages that may contribute to tumor progression, warranting functional evaluation in model systems that faithfully recapitulate DGC development and enable assessment of their therapeutic potential. In addition, integrative studies correlating DGC cellular morphology or disease stage with (epi-)genetic, proteomic, and transcriptomic data will be essential to further elucidate the mechanisms driving progression to advanced DGC.

## Supplementary Information

Below is the link to the electronic supplementary material.

Supplementary Material 1

Supplementary Material 2

Supplementary Material 3

Supplementary Material 4

Supplementary Material 5

Supplementary Material 6
