# Supplementary figures and images for: Mapping the genetic landscape of hereditary diffuse-type gastric cancer progression
Source: Gastric Cancer. 2026 Mar 27;29(3):527–39. doi: 10.1007/s10120-026-01730-1 (PMC13124845; doi:10.1007/s10120-026-01730-1)

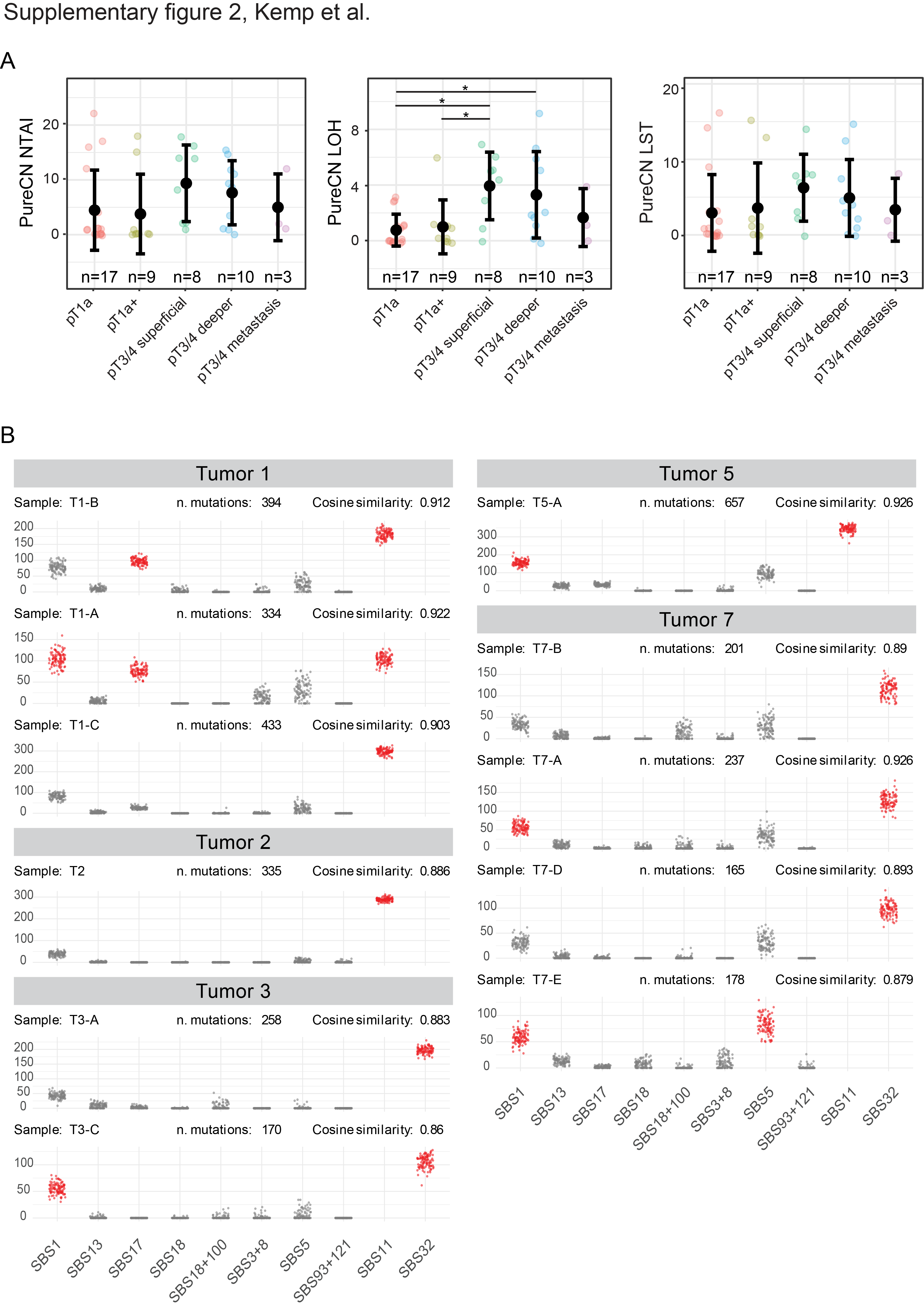

Supplement: Supplementary file 2 — Supplementary Material 2 [file 10120_2026_1730_MOESM2_ESM.tif]

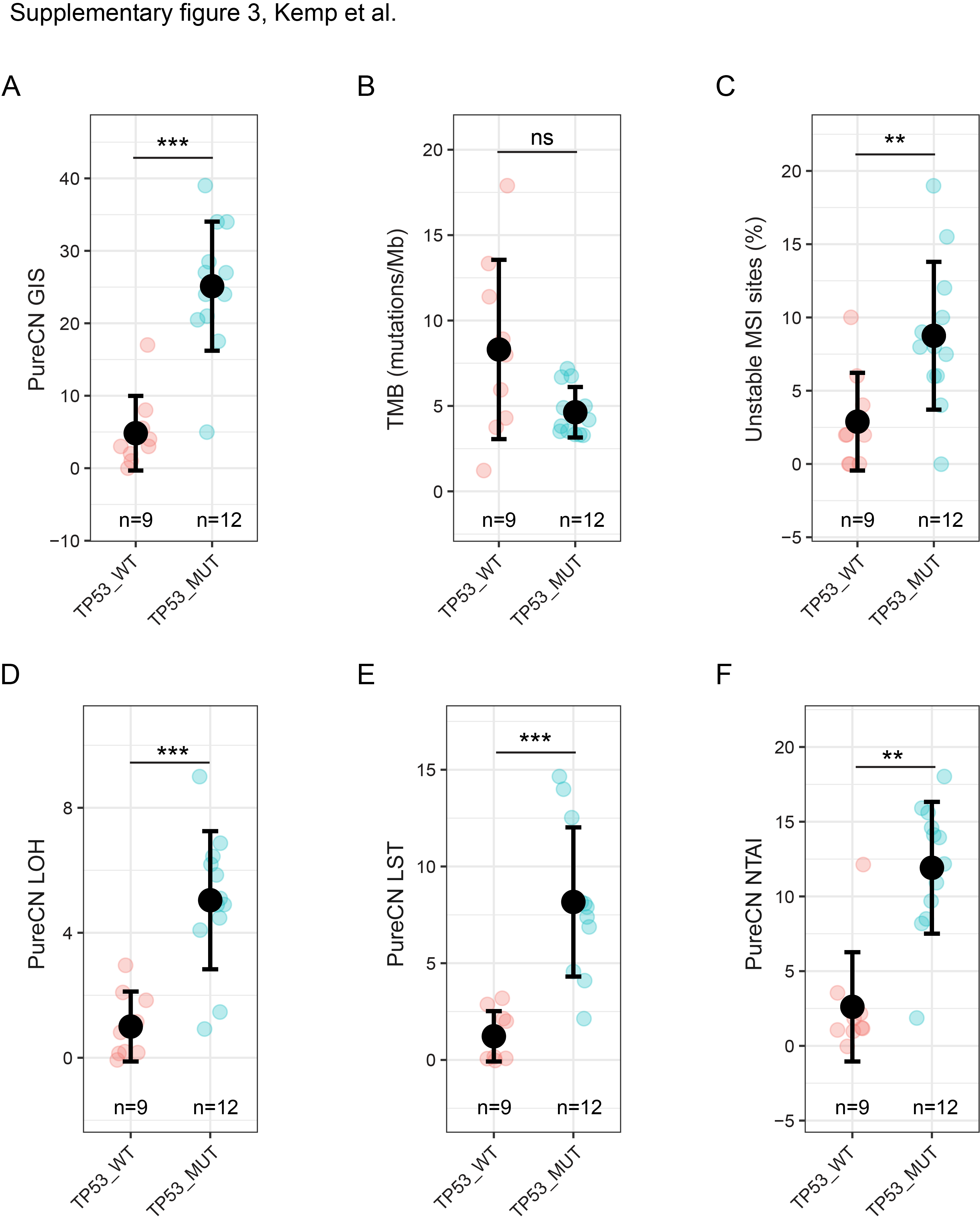

Supplement: Supplementary file 3 — Supplementary Material 3 [file 10120_2026_1730_MOESM3_ESM.tif]

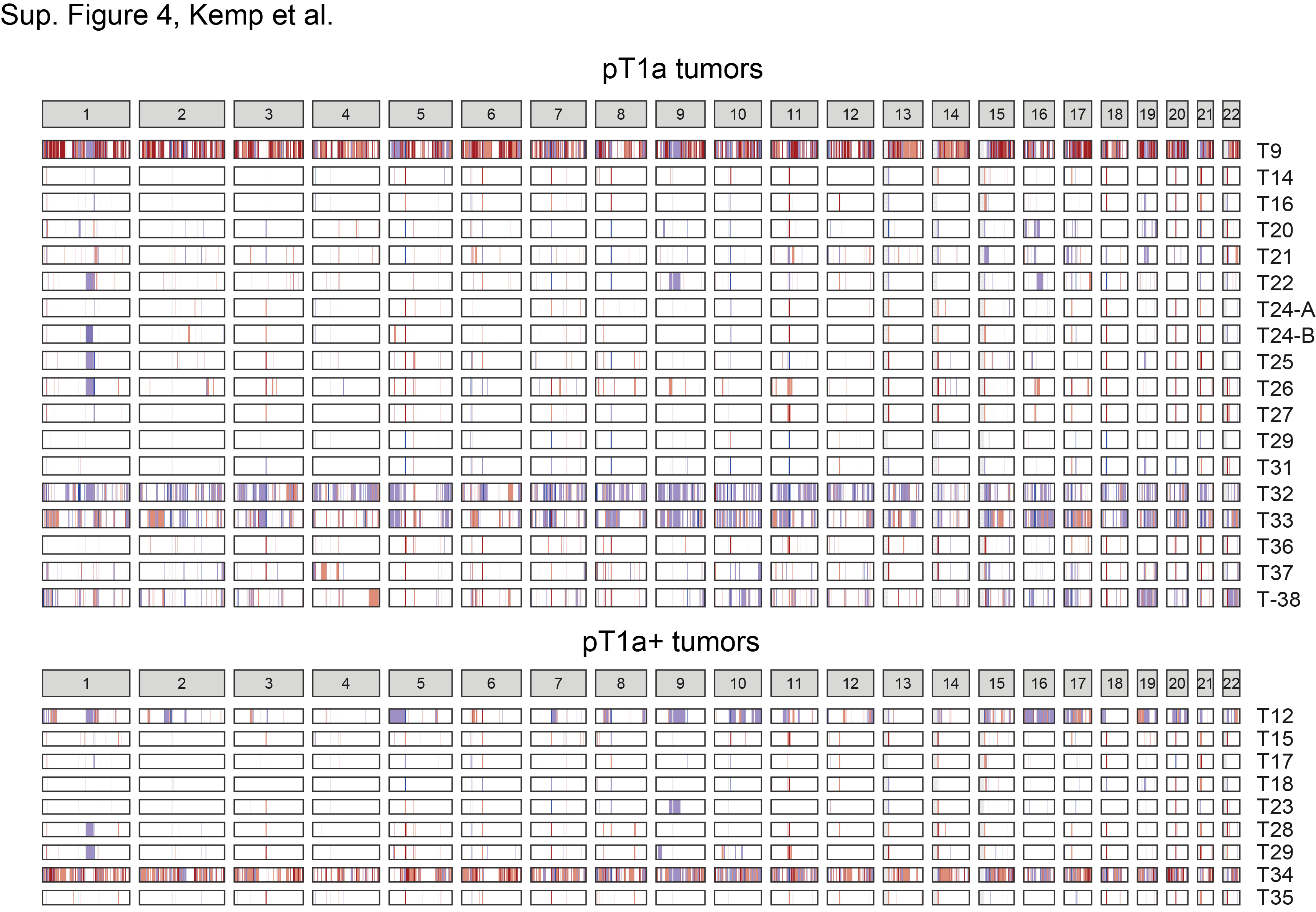

Supplement: Supplementary file 4 — Supplementary Material 4 [file 10120_2026_1730_MOESM4_ESM.tif]
