# Supplementary material for: Mapping the genetic landscape of hereditary diffuse-type gastric cancer progression
Source: Gastric Cancer. 2026 Mar 27;29(3):527–39. doi: 10.1007/s10120-026-01730-1 (PMC13124845; doi:10.1007/s10120-026-01730-1)
Supplement: Supplementary file 6 — Supplementary Material 6 [file 10120_2026_1730_MOESM6_ESM.docx]

# Supplementary Figure Legends

**Supplement Figure 1. E-cadherin expression patterns across HDGC lesions**

A) Representative H&E sections showing mucosal signet ring cell carcinoma and advanced diffuse gastric carcinomas (upper row) with corresponding E-cadherin immunohistochemical stains (lower row). For each distinct type of distribution (loss of E-cadherin expression, pink; weak to moderate membranous staining, yellow; and strong, membranous staining, teal) two representative examples are shown (130x magnification).

B) Quantification of the different types of E-cadherin expression patterns (see Fig S1A) across lesions of different stages of hereditary diffuse-type gastric cancer, showing the percentage of lesions with the indicated distributions of E-cadherin.

C) Quantification of the different types of E-cadherin expression patterns in pT1a and pT1a+ lesion, with connected lines indicating lesions analyzed within the same patient. Color codes are indicated in Fig. S1B.

### Supplementary figure 2. Genomic instability and mutational signature analysis

A) Dot plot showing the distribution of the separate values underlying the GIS-score (NTAI, LOH and LST) as determined by PureCN across different pathological stages of HDGC. Each dot represents the average score of a tumor region of an individual tumor. Dunn’s test, NTAI and LST all not significant. Dunn’s test of LOH, * p<0.05.

B) Signal signature analysis portraying the result of bootstrap refitting of signatures on mutations of individual samples (cosine > 0.85, and at least 150 mutations). Signatures that contribute for more than 20% to the mutational pattern of the sample are marked in red.

**Supplementary figure 3. Correlation genomic changes with *TP53* status**

A) Dot plot showing the GIS-score as determined by PureCN of *TP53*-wildtype and *TP53*-mutant advanced HDGC tumors. Each dot represents the average score of a tumor region of an individual tumor. Wilcoxon’s test, *** p<0.005.

B) Dot plot showing the Tumor Mutational Burden (TMB) of *TP53*-wildtype and *TP53*-mutant advanced HDGC tumors. Each dot represents the average score of a tumor region of an individual tumor. Wilcoxon’s test, ns = not significant.

C) Dot plot showing the percentage of unstable MSI sites as determined by PureCN of *TP53*-wildtype and *TP53*-mutant advanced HDGC tumors. Each dot represents the average score of a tumor region of an individual tumor. Wilcoxon’s test, ** p<0.01.

D) Dot plot showing the frequency of LOH as determined by PureCN of *TP53*-wildtype and *TP53*-mutant advanced HDGC tumors. Each dot represents the average score of a tumor region of an individual tumor. Wilcoxon’s test, *** p<0.005.

E) Dot plot showing the frequency of LST as determined by PureCN of *TP53*-wildtype and *TP53*-mutant advanced HDGC tumors. Each dot represents the average score of a tumor region of an individual tumor. Wilcoxon’s test, *** p<0.005.

F) Dot plot showing the frequency of NTAI as determined by PureCN of *TP53*-wildtype and *TP53*-mutant advanced HDGC tumors. Each dot represents the average score of a tumor region of an individual tumor. Wilcoxon’s test, ** p<0.01.

**Supplementary figure 4. Recurrent genomic aberrations in early HDGC lesions**

Visualization of the absolute total copy number values provided by PureCN of pT1a and pT1a+. Tumor samples T9, T12, T32, T33 and T34 exhibit focal amplifications and deletions scattered across the genome suggestive of analytical artefacts of PureCN associated with samples with lower tumor cell purity.

### Tables

**Table 1.** Clinical and Sample Characteristics of HDGC Cases Selected for Whole Exome Sequencing

M, male; F, female; CG, curative gastrectomy; PTG, prophylactic gastrectomy; Bx, biopsy; M, metastasis; N, normal; T, tumor; pTN-stage, pathological tumor-node stage; SL, superficial layer; DL, deeper layers; MET, metastasis; c., coding DNA; p., protein; del, deletion; dup, duplication; delins, deletion–insertion; fs, frameshift; *, stop codon.

# Supplementary tables

**Sup. Table 1** Overview of germline and somatic *CDH1*-mutations

**Sup. Table 2** LOH of *CDH1*

**Sup. Table 3** Somatic mutations in HDGC

**Sup. Table 4** Percentage occurrence mutations in early-stage HDGC

**Sup. Table 5** Percentage occurrence mutations in advanced-stage HDGC

**Sup. Table 6** Percentage occurrence mutations in sporadic DGC

**Sup. Table 7** Amplifications and deletions

**Sup. Table 8** List of genes with high variability

## **Supplementary Methods**

## Somatic variant calling

Tumor samples had a median coverage of at least 50x (range: 55.3x–934.3x). To ensure selection of high-confident somatic variants, variant calls were selected when they fulfilled the following criteria: sequencing depth >49 reads, >4 number of variant reads, variant allele frequency between >0.05 and <0.99, MPOS >7, and an orientation bias of F1R2 > 1 and F2R1 > 1. Subsequently, variants affecting the coding regions (including canonical splice-sites) were selected and germline variants common in the general European population (gnomAD European allele count heterozygous > 11, gnomAD European allele count homozygous > 11, gnomAD European allele frequency > 0.001) and a panel of normal samples from the RadboudUMC were excluded. Synonymous variants and variants with a CADD_Phred score < 15 were filtered out to prioritize variants predicted to have a higher functional impact. Variants were further filtered when they were present in normal tissues available of this cohort, except for variants in *CDH1*. Genes with known high variability (Supplementary Table 8) were excluded. Only variants in genes listed in the COSMIC cancer gene census database (version 102) were included for further analyses.^49^ Missense mutations were further classified using Franklin by Genoox (https://franklin.genoox.com, version 84.2), which incorporates *in silico* predictions, population data, and information of pathogenicity. For comparison to sporadic DGC, publicly available sequencing data of The Cancer Genome Atlas Pan-Cancer analysis project of 72 DGC samples (filtered on stomach; signet ring cell carcinoma or diffuse-type carcinoma)^50^ was obtained from the GDC portal using the TCGAbiolinks package in R.^51^ For defining *TP53* status (Supplementary Figure 3), 2 samples were considered mutant despite just falling below the selection critieria for somatic variant calling (T3-B, median coverage of 48 reads; T7-C, variant allele frequency of 0.048).

## Copy number plot

The absolute total copy number values provided by PureCN was used as input for visualization using cnSpec of GenVisR.

*Amplifications and deletions*

Significant tumor-specific amplifications (p-value < 0.001) were called by PureCN and deletions were called based on a relative gene coverage < 0.5 calculated by OncoCNV. Genes were retained if listed in the COSMIC Cancer Gene Census (v102) and excluded if they occurred in the list of highly variable genes (Supplementary Table 8).

## Mutational signature analysis

For mutational signature analysis, somatic mutations from tumors were selected based on single base substitutions (SBSs) with a variant allele frequency (VAF) of ≥5%. Samples containing at least 150 SBSs were analyzed using Signal.^52^ The resulting mutational profiles were subsequently filtered to retain only those with a cosine similarity >0.85 and a minimum signature contribution of 20%.

*Tumor mutational burden*

Tumor mutational burden (TMB) was calculated as the total number of variants (synonymous and non-synonymous) per megabase of callable exome regions.

**Supplementary References**

49. Sondka Z, Bamford S, Cole CG, Ward SA, Dunham I, Forbes SA. The COSMIC cancer gene census: describing genetic dysfunction across all human cancers. Nat Rev Cancer. 2018;18(11):696–705.

50.Weinstein JN, Collisson EA, Mills GB, Shaw KRMM, Ozenberger BA, Ellrott K, et al. The cancer genome atlas pan-cancer analysis project. Nat Genet. 2013;45(10):1113–20.

51. Colaprico A, Silva TC, Olsen C, Garofano L, Cava C, Garolini D, et al. TCGAbiolinks: an R/Bioconductor package for integrative analysis of TCGA data. Nucleic Acids Res. 2016;44(8):e71.

52. Degasperi A, Amarante TD, Czarnecki J, Shooter S, Zou X, Glodzik D, et al. A practical framework and online tool for mutational signature analyses show intertissue variation and driver dependencies. Nat Cancer. 2020;1(2):249–63.
